# Dismantling barriers to research and clinical care for individuals with a vision impairment

**DOI:** 10.5694/mja2.52627

**Published:** 2025-03-16

**Authors:** Eden G Robertson, Kate Hetherington, Meredith Prain, Julia Hall, Leighton Boyd AM, Rosemary Boyd OAM, Emily Shepard, Hollie Feller, Sally Karandrews, Fleur O'Hare, Kanae Yamamoto, Matthew P Simunovic, Robyn V Jamieson, Alan Ma, Lauren Ayton AM, Anai Gonzalez‐Cordero

**Affiliations:** ^1^ UNSW Sydney Sydney NSW; ^2^ Children's Medical Research Institute Sydney NSW; ^3^ Sydney Children's Hospitals Network – Randwick Sydney NSW; ^4^ Able Australia Melbourne VIC; ^5^ University of Melbourne Melbourne VIC; ^6^ Retina Australia Melbourne VIC; ^7^ UsherKids Australia Melbourne VIC; ^8^ Blind Citizens Australia Melbourne VIC; ^9^ Centre for Eye Research Australia Royal Victorian Eye and Ear Hospital Melbourne VIC; ^10^ Sydney NSW; ^11^ Save Sight Institute University of Sydney Sydney NSW; ^12^ Sydney Hospital and Sydney Eye Hospital Sydney NSW; ^13^ Sydney Children's Hospitals Network – Westmead Sydney NSW; ^14^ University of Sydney Sydney NSW

**Keywords:** Research design, Informed consent, Ethics, research, Quality of health care, Consumer health information, Healthcare disparities, Patient rights

In Australia, little prevalence data around vision impairment exist. However, self‐reported data from the Australian Bureau of Statistics 2017–18 National Health Survey^1^ suggest that around 800 000 people have a vision impairment or are blind (excluding uncorrected refractive errors).^2^ The leading cause of vision impairment in working‐age adults are inherited retinal diseases (IRDs)^3^ — a group of genetic conditions that primarily affect the retina. Other than one particular gene therapy for biallelic *RPE65*‐associated retinal dystrophy, there are no other clinically available treatments to safely prevent vision loss or restore vision for someone with an IRD.^4^

With no universally accepted definition of vision impairment,^5^ we use “vision impairment” in this article to refer to a significant reduction in vision that causes individuals to rely on visual substitution skills for daily living.^6^ Understanding the perspectives of individuals who have a vision impairment is necessary to develop meaningful interventions, policies and practices. However, too often these individuals have limited access to research opportunities and health information due to the inaccessibility of information.^7^, ^8^, ^9^ Resources, such as the *Web content accessibility guidelines*, provide useful guidance for how to make digital information more accessible.^10^, ^11^ However, a lack of awareness and integration of these guidelines across national policy and professional code results in little uptake. Challenges around feasibility and capacity also arise for researchers and clinicians when embedding such practices.

## Sharing our experiences

In 2023–2024, we undertook a James Lind Alliance Priority Setting Partnership (PSP) to identify the top research priorities for IRDs in Australia, from the perspectives of individuals with lived experience and health professionals.^12^, ^13^ As a first step of the PSP, we established a 14‐member steering group consisting of people with lived experience (individuals with an IRD or other vision impairment, and caregivers), community organisations representatives, health professionals (clinical geneticists, ophthalmologists, speech pathologist, optometrist), and researchers. The steering group met ten times over 18 months to guide the PSP, which included two national surveys and online workshops with the IRD community.^12^

Here, we share our learnings from undertaking the PSP surveys and hosting an in‐person educational event for the IRD community. This article has been co‐written with our PSP steering group, including seven co‐authors who have lived experience of vision impairment. By providing these co‐developed recommendations, summarised in the Box, our aim is to provide feasible strategies for researchers and clinicians to undertake more accessible research and better facilitate information access for individuals with a vision impairment.

Box 1 **Recommendations to improve information access for people who have a vision impairment**

**Table:** 

**Build meaningful connections with individuals who have lived experience and community organisations**
Establish a committed steering committee comprising individuals who are trusted in the community and connected to your target audience Allocate appropriate time and resources to develop partnerships Provide communication plans and promotional material for partners to use when supporting recruitment Implement a study newsletter to keep partners and steering group members engaged and feeling valued
**Shift the focus to people‐first language and a social model of disability**
Use people‐first language, with good practice to ask each individual their preferred terminology Underpin research practices with the social model of disability Invite individuals with lived experience to contribute to the research team as equal partners
**Implement strategies to ensure all individuals can fully participate**
Be proactive in providing a list of available supports and considerations to each participant before an event Invite participants to share any accessibility requirements that will allow them to fully participate Allocate appropriate time in meetings for written content to be read out loud Send any content shared in meetings at least one week in advance. When in a group setting: ‣ ensure the facilitator is appropriately trained to work with this cohort; ‣ invite speakers to announce their name before speaking; ‣ use verbal social cues as needed; and ‣ avoid the use of the “raise hand” or chat function if meeting online When meeting in person: ‣ assign staff to provide navigation support, with descriptive directions of the environment, location, and navigation supports that will be provided, and share this information before the event; ‣ design the physical space to ensure comfortable navigation with a guide dog, white cane, and/or support person; and ‣ offer attendees access to an audio induction loop
**Move away from the default methods of information provision**
Create video explainers, with spoken English and written transcriptions (ie, not just subtitles) Minimise use of visuals that are unnecessary, embedding alt text for any necessary visuals Provide written information in multiple formats (ie, digital copies and other modes such as audio) that are compatible with screen readers, can be zoomed in on, and with a QR code for online access Implement accessibility recommendations (eg, font size) from relevant consumer organisations into written documents and presenter templates (Supporting Information) Use freely available accessibility checkers when creating Word or PDF documents Ensure that all punctuation is provided to ensure that screen readers pick up the text correctly Consider using dot points to provide breaks in text
**Adapt data collection methods**
User‐test data collection methods with several individuals with lived experience, incorporating their feedback as appropriate Provide the option for participants to request an alternative format and/or language for study participation Allocate a budget for providing an alternative format and/or language for study participation (eg, Braille) in case participants request adaptations

### Build meaningful connections with individuals who have lived experience

Our steering group was integral to ensuring an inclusive study design and casting a wide enough recruitment net to ensure diversity of respondents. They supported recruitment through leveraging of personal networks and connections with over 20 community‐based organisations who also promoted our PSP surveys via mechanisms such as social media posts and member newsletters. We invited organisations to partner given their experience communicating with their specific communities, direct access to our target audience and established status as trusted sources of information. We provided suggested content and social media tiles to reduce the burden on these often limited‐staffed organisations who provided in‐kind support.

### Shift the focus to people‐first language and a social model of disability

Our research practices veered away from the medical model of disability toward a social model, ensuring that individuals with lived experience were equal partners with the researchers. At study outset, we acknowledged the researchers’ responsibility in making necessary accommodations to support their meaningful participation.

Although differences exist within and between communities,^14^ the use of people‐first language (ie, highlighting the individual person first, rather than their disability) was mentioned several times by our steering group as their preference. For our PSP and event, we used people‐first language. However, this was an experiential learning curve, with resources requiring updates as we gained further insights from the community, and with the ongoing debate around appropriate language within the broader disability sector. We also minimised the use of the term “patient” to only denote a person who was receiving medical care, rather than as a collective term to refer to people living with an IRD. Following good practice, we remained flexible by continuing to ask for preferences and to mirror the language of individuals with lived experience.^15^

### Implement strategies to ensure all individuals can fully participate

Before the first steering group meeting, we invited members to share any needs or preferences to be able to fully participate in meetings. This resulted in numerous accommodations, such as members announcing their name before speaking. Allocated meeting times also took into consideration that written content shared on screen would be read out loud.

In preparing our event, we prioritised the importance of lighting, physical navigation space, audio induction loops, and location. We invited attendees to share any accessibility requirements when registering, and communicated about the space to attendees ahead of time. When opening the event, our Master of Ceremonies (a person who has a vision impairment) also provided descriptive directions of the space. For our lived experience panel, the Panel Chair (a person who has a vision impairment) directed questions to specific individuals in the order of their seated position, which ensured conversation flow without the use of non‐verbal, turn‐taking cues.

### Move away from the default methods of information provision

Videos and infographics are effective tools for sharing health information. However, relying solely on these formats can discriminate against individuals with vision impairment. For our PSP, we developed a short animation that also verbally explained the study. Following feedback from partners about our first PSP survey around eligibility, we also developed a video about what IRDs are. Developing this in Auslan as the primary language (to support a proportion of the IRD community who are deafblind), complemented with spoken English and a transcription, made it more accessible without detriment to sighted and hearing populations.^16^

For our PSP and event, we developed materials following accessibility recommendations from Vision Australia^17^ and VisAbility,^18^ and included QR codes for online access if provided in hard copy. All materials could be accessed via screen readers and could be magnified. We underwent multiple reviews of formatting and punctuation, especially full stops, which is crucial for people using screen readers. See the Supplementary Information for our co‐developed Word template.

We provided our event presenters with guidelines and PowerPoint templates. Presenters were advised to minimise the use of PowerPoint presentations and to provide verbal descriptions of any necessary visuals.

### Adapt data collection methods

For our PSP surveys, we used the online survey platform Qualtrics (www.qualtrics.com/en‐au) as our primary data collection method because it was deemed by our steering group as compatible with most screen readers. We undertook numerous rounds of user testing with individuals of different ages and levels of comfort with technology. We also provided the opportunity for participation on paper (including in Braille), phone call (with the option for a language interpreter), or video teleconference in Auslan. Participation in the more than 220 survey responses received was predominantly in written English via Qualtrics or paper. As such, we recommend that alternative approaches to data collection be scoped and budgeted should they be requested, rather than investing significant efforts at project outset.

## Conclusion

Accessibility is an ongoing commitment that will continue to change based on the needs and experiences of the community. Here, we share our current recommendations for more equitable access to research and clinical information for individuals who have a vision impairment. We strongly suggest that researchers and clinicians weigh up the potential value and impact of incorporating these recommendations with the social and ethical cost of forgoing them.

## Open access

Open access publishing facilitated by University of New South Wales, as part of the Wiley – University of New South Wales agreement via the Council of Australian University Librarians.

## Competing interests

No relevant disclosures.

## Provenance

Not commissioned; externally peer reviewed.

## Supporting information


Supplementary template
