# Supplementary material for: Dismantling barriers to research and clinical care for individuals with a vision impairment
Source: Med J Aust. 2025 Mar 16;222(7):324–6. doi: 10.5694/mja2.52627 (PMC12009595; doi:10.5694/mja2.52627)
Supplement: Supplementary file 1 — Supplementary template [file MJA2-222-324-s001.pdf]

The Medical Journal of Australia

Supporting Information

Supplementary material

This appendix was part of the submitted manuscript and has been peer reviewed. It is posted as supplied by the authors.

Appendix to: Robertson EG, Hetherington K, Prain M, et al. Dismantling barriers to research and clinical care for individuals with a vision impairment. *Med J Aust* 2025; doi: 10.5694/mja2.52627.

Word template

This Word template provides a useful starting point when creating resources for people who have a vision impairment. Depending on purpose, this may require further changes to best suit the needs of your cohort. User-testing with your primary cohort is highly recommended.

Font

- For Word or PDF documents, minimum font size of 14.
- Minimum line spacing of 1.5.
- Avoid special fonts (e.g., Italics or stylised fonts) and fully capitalised words.

Format

- Be consistent with the alignment of text, preferably being left-aligned.
- Consider using bullet points to break up the text. Try to start each bullet with a different word or use numbered bullets.
- Maintain normal margins.
- Include page numbers.
- Tables should be used to convey data and not as an alternative way of organising the text to make it more visually interesting.

Example 1. Table that is used correctly.

**Count**	**2021**	**2022**
NSW	1,172	1,433
Victoria	1,468	2,054

Example 2. Table that is used incorrectly.

Diagnosis	Age	Gender
Postcode	Children	Education

Colours

- High contrast colours (e,g., black text on a white background)
- Use of dark background and light text may be preferable if you anticipate there to be extended periods of viewing.

Example 1. High contrast

Hello

Example 2. Low contrast

Hello

Visuals

- Embed alt text for any visuals.
- If using visuals, ensure it is of high colour contrast and can be magnified 200% without impacting the quality.

[Insert image]

Embed Alt Text: 1-2 sentences describing the image

View this document online via the QR code below.
[insert QR code]
